# Dendritic cells trigger imbalance of Th1/Th2 cells in silica dust exposure rat model *via* MHC-II, CD80, CD86 and IL-12

**DOI:** 10.1039/c8ra03970d

**Published:** 2018-07-20

**Authors:** Lei Bao, Changfu Hao, Suna Liu, Lin Zhang, Juan Wang, Di Wang, Yiping Li, Wu Yao

**Affiliations:** School of Public Health, Zhengzhou University No. 100 Science Avenue Zhengzhou Henan 450001 China yaowu@zzu.edu.cn +86-371-67781922 +86-371-67781922; The Third Affiliated Hospital of Zhengzhou University Zhengzhou Henan 450001 China; Hebei General Hospital Shijiazhuang Hebei 050000 China

## Abstract

Silicosis is one of the most common occupational respiratory diseases caused by inhaling silica dust over a prolonged period of time, and the progression of silicosis is accompanied with chronic inflammation and progressive pulmonary fibrosis, in which dendritic cells (DCs), the most powerful antigen presentation cell (APC) in the immune response, play a crucial role. To investigate the role of DCs in the development of silicosis, we established an experimental silicosis rat model and examined the number of DCs and alveolar macrophages (AMs) in lung tissues using immunofluorescence over 84 days. Additionally, to obtain an overview of the immunological changes in rat lung tissues, a series of indicators including Th1/Th2 cells, IFN-γ, IL-4, MHC-II, CD80/86 and IL-12 were detected using flow cytometry and an enzyme-linked immunosorbent assay (ELISA) as well as a real-time polymerase chain reaction (PCR) assay. We observed that the number of DCs slightly increased at the inflammatory stage, and it increased significantly at the final stage of fibrosis. Polarization of Th1 cells and IFN-γ expressions were dominant during the inflammatory stage, whereas polarization of Th2 cells and IL-4 expressions were dominant during the fibrotic stage. The subsequent mechanistic study found that the expressions of MHC-II, CD80/86 and IL-12, which are the key molecules that connect DCs and Th cells, changed dynamically in the experimental silicosis rat model. The data obtained in this study indicated that the increase in DCs may contribute to polarization of Th1/Th2 cells *via* MHC-II, CD80/86, and IL-12 in silica dust-exposed rats.

## Abstract 2

Dendritic cells (DCs), the most powerful antigen presentation cell (APC) in the immune response, play a crucial role in silicosis.

## Graphical abstract

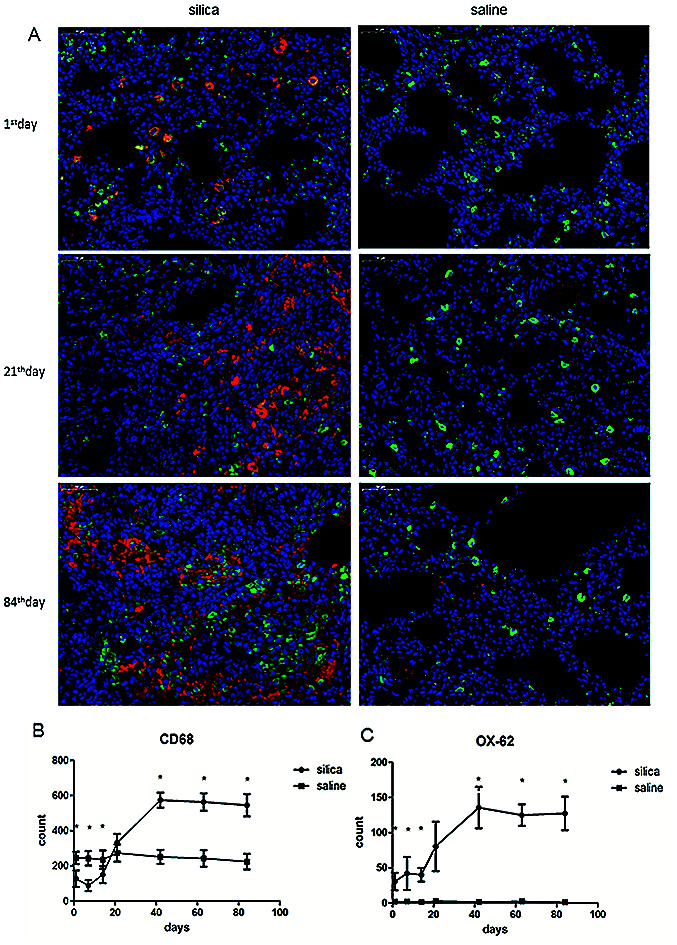

## 1 Introduction

Silica dust is one of the most common environmental and occupational risk factors; long term exposure to silica dust contributes to the development of a number of diseases including silicosis, systemic lupus erythematosus (SLE) and rheumatoid arthritis (RA).^1^ Due to lack of widely accepted criteria for diagnosis or classification of autoimmunity and animal models that mimic silica dust exposure in humans, studies related to the diseases caused by silica dust have always focused on silicosis.^2,3^ Silicosis is a fibrotic lung disease caused by the inhalation of silica dust. Occupational exposure to respirable silica particles occurs in many situations, which are often called the dusty trades and include abrasive blasting with sand, jack hammering, drilling, mining/tunneling operations, and cutting and sawing.^4^ The prevalence of disorders associated with silica dust exposure is widely observed, especially in low and middle income countries, where actual cases are often under-reported because of poor surveillance.^5^ To date, the pathogenesis of silicosis is still unclear. Numerous studies have proposed several mechanistic hypotheses, which are not systematic and remain tentative.^6–9^ The immune hypothesis has been confirmed with high consensus in silicosis research.^10^

Silicosis is a complex immune process including the identification, uptake and presentation of silica dust, which triggers and regulates the immune response through mechanisms that have not been established. The Si–OH complex of silica and H_2_O is similar to pathogen-associated molecular patterns (PAMPs) and therefore, it is recognized and bound by pattern recognition receptors (PRRs) on AMs;^11^ it can induce AMs to secret cytokines and chemokines to initiate the influx of inflammatory cells such as macrophages, neutrophils, and lymphocytes.^12,13^ Upon activation of the innate immune system, silicosis presents an acute inflammatory reaction at an early stage, which is characterized by infiltration of inflammatory cells and destruction of alveolar walls. Accompanied by the activation of inflammatory cells and the secretion of cytokines and chemokines, adaptive immunity is also involved in the development of silicosis. Many fibroblasts are activated and proliferated, releasing a large amount of collagen; silicosis progresses to diffuse interstitial fibrosis or eventually form silicotic nodules.^14,15^ Previous studies have demonstrated that CD4^+^ T cells are considered as the key participant in silicosis, and Th1/Th2 cells participate in the pathogenesis of silicosis.^16,17^

DCs are the most efficient APCs that can activate both T cells and B cells and thus, they act as a bridge between innate and adaptive immunity.^18–21^ Besides, DCs also have the ability to influence T cell polarization *via* three ways: (i) antigen presentation, (ii) co-stimulatory molecule expression, and (iii) direct contribution by DCs to the immediate cytokine milieu that directs the resultant Th cell response.^22^ MHC-II, CD80, CD86, and IL-12 have been shown as key molecules that connect DCs and Th cells in immunological diseases.^23–25^ In addition, CD86 and IL-12 are crucial for Th1 priming, whereas no exact mechanism for the regulation of Th2 exists.^26,27^ Recent studies have indicated that DCs are associated with fibrotic diseases.^28–30^ Studies on human fibrotic interstitial lung diseases have also demonstrated that the resident cells in pulmonary fibrosis can sustain chronic inflammation by driving the accumulation of DCs with the potential to mature locally within ectopic lymphoid follicles.^31^ However, there is no relevant study on the regulation of Th1/Th2 cell polarization by dendritic cells in silicosis.

This study was designed to determine whether silicosis is associated with DCs using the rat model of experimental silicosis; it was also designed to know whether polarization of Th1/Th2 cells by DCs is involved. Herein, we characterized the number of DCs and examined Th1/Th2 cells and the expression of cytokines up to 84 days to assess potential mechanisms of silicosis.

## 2 Materials and methods

### 2.1 Experimental animals

Male Sprague-Dawley (SD) rats (age: 6–8 weeks; weight: 180–220 g) were purchased from the Laboratory Animal Center of Henan Province (Zhengzhou, China). All rats were kept at the Zhengzhou University specific-pathogen-free (SPF) laboratory animal facility. Cages, bedding, and food were sterilized by autoclaving. All experimental procedures were performed in strict accordance with “Principles of Laboratory Animal Care and Use in Research” (State Council of China, 1988) and were approved by the Institutional Animal Care and Use Committee of Zhengzhou University (Zhengzhou, China).

### 2.2 Generation of animal silicosis model

Eighty-four rats were divided into treatment and control groups; 6 from each group were euthanized on 1^st^, 7^th^, 14^th^, 21^th^, 42^th^, 63^th^ and 84^th^ day post injection. Silica (SiO_2_ purity >99%, average particle size 0.5–10 μm, Sigma-Aldrich, Shanghai, CN) was ground and dried. Particulates were suspended in sterile saline at a concentration of 100 mg ml^−1^. Prior to endotracheal instillation, penicillin (North China Pharmaceutical Co. Ltd., Shijiazhuang, CN) was added at 2000 units per ml. The rats were anesthetized with ether and then were hung on the metal shelf by hooking the string with their teeth. Endotracheal intubation was performed when the tracheal opening was seen from the rat's mouth. After successful intubation, 1 ml of SiO_2_ suspension was rapidly pushed into the trachea and then, 2 ml of air was pushed into the trachea. The intubation tube was rapidly pulled out, and the suffocation was lifted as soon as possible. Saline exposure rats were injected with the same volumes of sterile saline and penicillin in the same manner.

### 2.3 Euthanization procedure

Rats were euthanized with a sealed euthanasia device, which had good transparency and a convenient window to observe whether the animal died or not. Before the rats were placed into the device, we put a certain amount of carbon dioxide into the device, so that the rats could enter anesthesia faster with reduced fear and pain. Moreover, carbon dioxide was continuously passed for 2 to 3 minutes after the rats were euthanatized.

### 2.4 Immunofluorescence (IF)

The expressions of CD68 and OX-62, which are specific biomarkers of AMs and DCs, respectively, were observed in lung tissues using double-labeling immunofluorescence.^32–34^ All rats were sacrificed by luxation of cervical vertebra to collect the same section of the right lung tissue, which was then fixed in 10% neutral formalin and embedded in paraffin. Paraffin sections were deparaffinized, rehydrated in xylene and ethanol and then treated with 3% H_2_O_2_ (Boster Biological Technology, Ltd, Wuhan, CN) for 10 min to suppress endogenous peroxidase activity and reduce background staining. After heating in citrate buffer (Boster Biological Technology) for 20 min, the sections were blocked with 10% goat serum (Boster Biological Technology) in TBS for 1 hour at room temperature. These sections were then incubated overnight at 4 °C with mouse anti-rat OX-62 (dilution 1 : 50, BD Pharmingen, San Jose, CA, USA) and rabbit anti-rat CD68 (dilution 1 : 200, Abcam, USA) for immunofluorescent double staining. Next, the sections were incubated with TRITC goat anti-mouse IgG for mouse anti-rat OX-62 (dilution 1 : 400, Boster Biological Technology) and FITC goat anti-rabbit IgG for rabbit anti-rat CD68 (dilution 1 : 400, Boster Biological Technology) and mounted under coverslips, sealed with nail polish to prevent drying and movement under the microscope. We randomly selected 5 visual fields (×400) for each slice; positive staining for OX-62 was indicated by red staining, and CD68 positivity was indicated by green staining. The Image-Pro Plus 6.0 software was used to analyze the number of positive cells in each photo.

### 2.5 Real-time PCR

The sequences of MHC-II, CD80, CD86 and IL-12 genes were retrieved from the GenBank of the National Center for Biotechnology Information (NCBI) database (Table 1). Rat lungs were minced and digested in RPMI 1640 (Gibco, Grand Island, NY, USA) with 2 mg ml^−1^ collagenase D (Roche Diagnostics GmbH, Mannheim, Germany) for 30 min at 37 °C, and single-cell suspensions were prepared. The cells were incubated with rat anti-DC (OX-62) microbeads (Miltenyi Biotec, CA, USA) at 4 °C for 15 min, and positive selection was performed with a MidiMACS Starting Kit (Miltenyi Biotec, CA, USA). RNA extraction was performed on OX-62^+^ DCs using the Tri Reagent procedure according to the manufacturer's instructions (Sigma-Aldrich, Shanghai, CN). Reverse transcription was performed with 2 μg of total RNA, 10 μM random primers, 2.5 nM dNTPs, 0.1 M DTT, 20 U RNAse-OUT (Invitrogen, Waltham, MA, USA) and RNase-free water. This reaction mixture was incubated at 37 °C for 40 min, followed by enzyme inactivation at 70 °C for 15 min. cDNA samples were stored at −20 °C until further processing. Real-time PCR was performed in the StepOnePlus real-time PCR system (Applied Biosystems, Foster City, CA, USA) using a FastStart Universal SYBR Green Master (Rox) Kit (Roche Diagnostics GmbH, Mannheim, Germany). Real-time RT-PCR was performed in a final volume of 10 μl containing 5 μl of 2× SYBR Green Master Mix, 0.25 μl each of 0.5 μM F1 forward and R1 reverse primers, 2.5 μl of cDNA or standard plasmid DNA, and 2 μl of ultrapure water. The optimized thermal cycling conditions were as follows: 95 °C for 10 min, 95 °C for 15 s, and 60 °C for 60 s for 40 cycles.

**Table 1:** The primer sequences of genes of CD80, CD86, MHC-II and IL-12

Gene name	Forward primer	Reverse primer
MHC-II	GTTGGTGATGCTGGAGATGGTT	GCAGACGTGGACTGTGCTTTC
CD80	CAGGTTCATTCATCTCTTTGTGC	GACAGCAATGCCTTTTCTCTCAC
CD86	AGGACACGGGCTTGTATGATTG	GGTTTCGGGTATCCTTGCTTAG
IL-12	AAGGTCACACTGAACCAAAGG	TGATGTCCCTGATGAAGAAGC

### 2.6 Flow cytometry

Lungs were minced and incubated in RPMI-1640 (Gibco, Grand Island, NY, USA) with 2 mg ml^−1^ collagenase D for 30 min in 37 °C. After digestion, the lung cells were dispersed by gentle pipetting, followed by filtration through a 75 μm cell strainer. Then, 20 ng ml^−1^ of phorbol 12-myristate 13-acetate and 1 μg ml^−1^ of ionomycin (Sigma-Aldrich) were added to the cells. The cells were incubated at 37 °C in 5% CO_2_ for 1 hour and then supplemented with 10 μg ml^−1^ Brefeldin A (Sigma-Aldrich, Shanghai, CN) and incubated for another 4 hours. The cells were resuspended in 100 μl of staining buffer containing FITC-labeled mouse anti-rat CD3 and PerCP mouse anti-rat CD8a (BD Pharmingen) antibodies, and they were incubated for 30 minutes at 4 °C, fixed and permeabilized by addition of 500 μl of fixation/permeabilization solution (BD Pharmingen), vortexed and then incubated at RT in the dark for another 20 minutes. Subsequently, the cells were resuspended in 100 μl of Perm/Wash buffer containing PE mouse anti-rat IL-4 and Alexa Fluor® 647 mouse anti-rat IFN-γ antibodies and stained for 30 minutes at 4 °C. Mouse IgG1 PE isotype control (BD Pharmingen) and mouse IgG1 Alexa Fluor® 647 isotype control (BD Pharmingen) were used as negative controls. All samples were washed, resuspended in 2% paraformaldehyde and analyzed by flow cytometry (Accuri C6, BD Accuri).

### 2.7 Cytokine enzyme-linked immunosorbent assay (ELISA)

A total of 100 mg of the same sections of the rat lung tissue, previously used for the immunofluorescence study, was ground on ice. The tissue samples were then centrifuged, and the resulting supernatant was collected and analyzed for IFN-γ and IL-4 production using murine cytokine ELISA kits according to the manufacturer's protocol (Boster Biological Technology).

### 2.8 Statistical analysis

All data were collected and analyzed using Microsoft Excel 2013 (Microsoft, Redmond, WA, USA) and SAS 9.2 for windows (SAS Institute Inc., Cary, NC, USA) respectively, and the values of continuous variables were expressed as mean ± standard error of mean (SEM). Differences between any two independent samples under normal distribution were compared using Student's *t*-test. A *P* value less than 0.05 was considered as statistically significant unless otherwise indicated.

## 3 Results

### 3.1 Characterization of pathological changes of lung tissue from silicotic rats

**Fig. 1:**
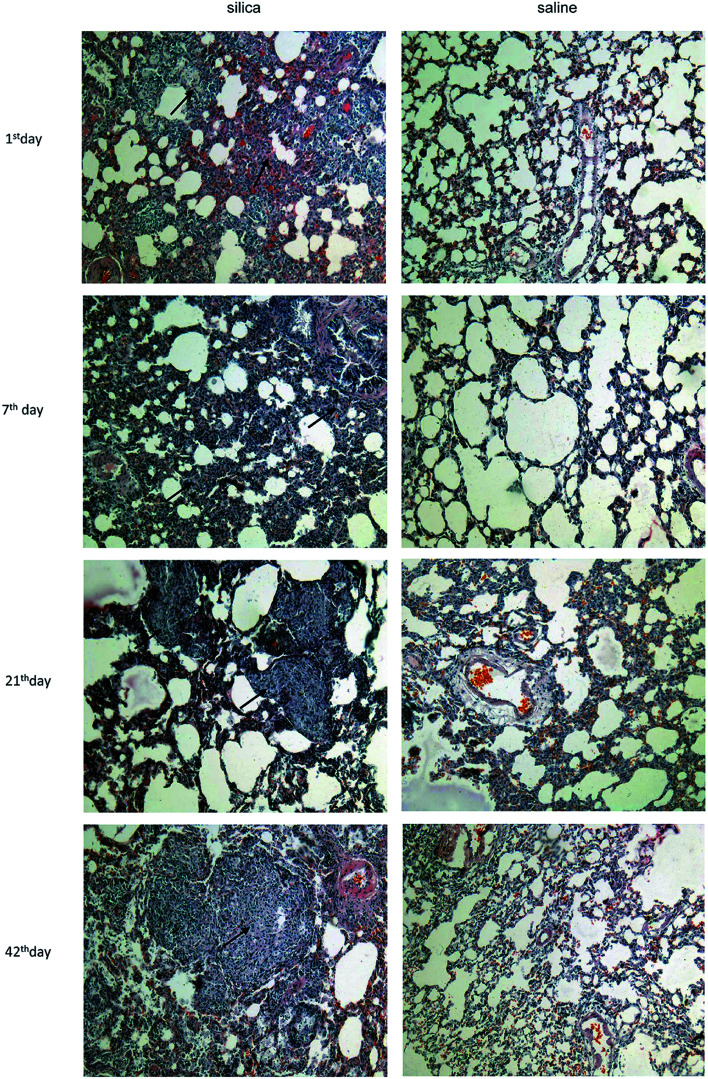
Characterization of pathological changes in lung tissue from silicotic rats. Lung sections were stained with hematoxylin and eosin (magnifications ×100).

Clear infiltration of inflammatory cells and a thickened alveolar septum were observed on the 1^st^ day and 7^th^ day post injection. On the 21^th^ day, several nodules with increased cellularity and some fibrotic nodules were observed among lung tissues, blood vessels and bronchus. Interstitial edema, haemorrhage and infiltration of inflammatory cells such as macrophages and lymphocytes were observed in interstitial nodules. Many cellular nodules, fibrotic nodules and fused pulmonary alveoli were observed on 42^th^, 63^th^ and 84^th^ days, and considerable fusion was found among gaps of nodules and fractures of the alveolar septum (Fig. 1).

### 3.2 Response of AMs and DCs to silica dust exposure

**Fig. 2:**
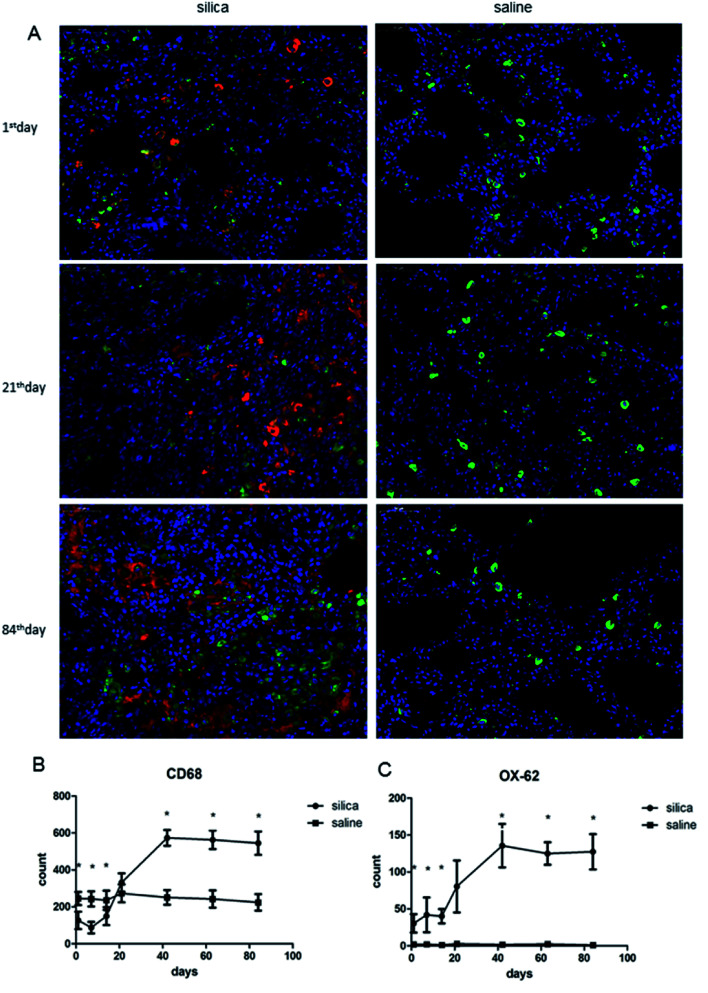
Double-labeling immunofluorescence of DCs and AMs in lung tissues of rats with or without dust exposure over 84 days (A). Detection of DCs and AMs using OX-62 (red) and CD68 (green), respectively (original magnification, ×400). Changes in the number of AMs (B). Changes in the number of DCs (C). *n* = 6. Error bars indicate the SEM. *P < 0.05 compared with the saline group. All values represent the mean ± SEM.

We performed double-labeling immunofluorescence on DCs and AMs in lung tissue sections (Fig. 2A). The number of AMs significantly decreased in rats from the 1^st^ day of silica dust exposure and reached the lowest level on the 7^th^ day; then, it increased and returned to the normal level on the 21^th^ day. The level of AMs continued to increase and reached the highest level on the 42^th^ day, and this level was maintained till the end of the observation. The difference in AM numbers between silica dust exposure rats and control was statistically significant at each time point except for the results of the 21^st^ day (*P* < 0.05); the number of DCs increased from the 1^st^ day, and even more DCs were observed on the 42^th^ day in the silica dust exposure rats (*P* < 0.05). These data demonstrated that the number of AMs and DCs changed upon exposure to silica dust (Fig. 2B and C).

### 3.3 Expression of Th1/Th2 cells and cytokines response to silica dust exposure

**Fig. 3:**
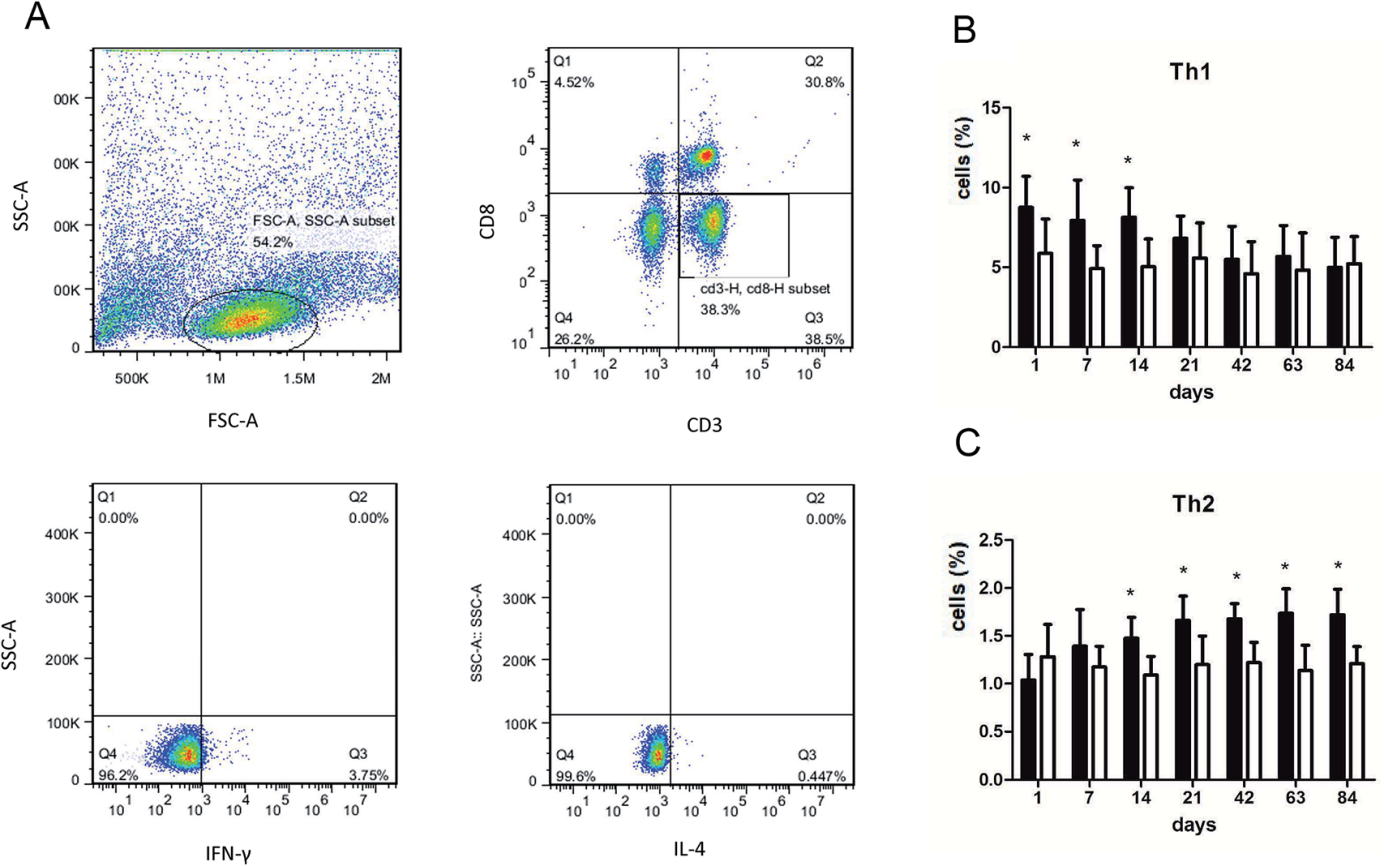
Changes of Th1/Th2 cells in lung tissue of rats with or without dust exposure over 84 days. Flow cytometry analysis of intracellular IFN-γ and IL-4 expressions in CD4^+^ T cells from rat lungs (A). Changes in percentage of Th1 cells (B). Changes in percentage of Th2 cells (C). *n* = 6. Error bars indicate the SEM. *P < 0.05 compared with the saline group. All values represent the mean ± SEM.

**Fig. 4:**
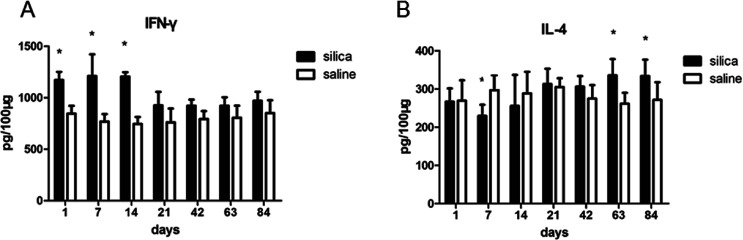
Changes in cytokines in lung tissue of rats with or without dust exposure over 84 days. Changes in expression of IFN-γ (A). Changes in expression of IL-4 (B). *n* = 6. Error bars indicate the SEM. *P < 0.05 compared with the saline group. All values represent the mean ± SEM.

We performed flow cytometry analysis to detect intracellular IFN-γ and IL-4 expressions in CD4^+^ T cells in single cell suspensions of lung tissues (Fig. 3A). The subsets of Th cells in silica dust exposure rats changed remarkably compared to those of the controls. The proportion of Th1 cells in the silica dust exposure rats increased rapidly after exposure to silica; then, it decreased gradually with dust time. The difference between silica dust exposure rats and the control was statistically significant on 1^st^, 7^th^ and 14^th^ days (*P* < 0.05) (Fig. 3B). Inversely, the proportion of Th2 cells in silica dust exposure rats increased gradually with the increase in dust exposure time; except on 1^st^ and 7^th^ day, the difference between silica dust exposure rats and control was statistically significant (*P* < 0.05) (Fig. 3C). We therefore performed ELISAs to detect IFN-γ and IL-4 in lung tissue, and we found that Th1 cells and IFN-γ as well as Th2 cells and IL-4 had similar trends. IFN-γ expression increased during the inflammatory stage (*P* < 0.05) (Fig. 4A), whereas IL-4 expression increased in the fibrotic stage (*P* < 0.05) (Fig. 4B). Based on these findings, it was apparent that the polarization of Th1/Th2 cells exists in silica dust exposure rats, which may contribute to the development of silicosis.

### 3.4 Costimulatory molecules in OX-62^+^ DCs that regulate Th1/Th2 cells

**Fig. 5:**
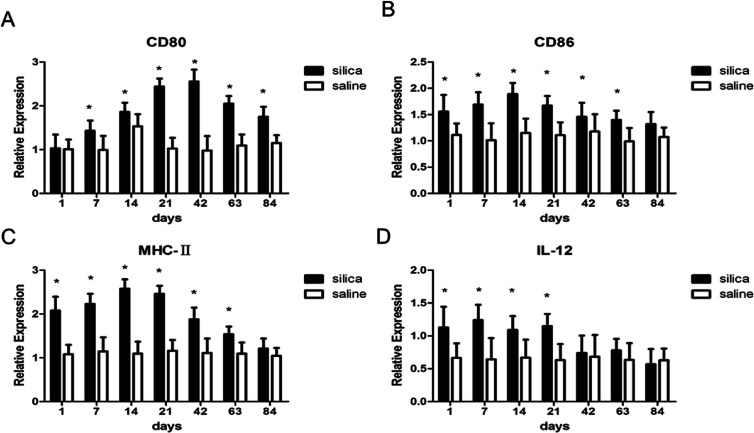
Expressions of MHC-II, CD80, CD86 and IL-12 in lung tissue of rats with or without dust exposure over 84 days. Changes in expression of CD80 (A). Changes in expression of CD86 (B). Changes in expression of MHC-II (C). Changes in expression of IL-12 (D). *n* = 6. Error bars indicate the SEM. *P < 0.05 compared with the saline group. All values represent the mean ± SEM.

To investigate whether DCs could regulate Th1/Th2 cells, the costimulatory molecules CD80, CD86, MHC-II and IL-12 in OX-62^+^ DCs were detected using real-time PCR. The results showed elevated gene expressions for MHC-II, CD80, CD86 and IL-12 in silica dust exposure rats. The difference in expression for CD80 at all time points except on 1^st^ day, for CD86 and MHC-II at all time points except on 84^th^ day, for IL-12 on 1^st^, 7^th^, 14^th^, and 21^th^ day between silica dust exposure rats and control was statistically significant (*P* < 0.05) (Fig. 5). These results confirmed that DCs may regulate Th1/Th2 cells by these costimulatory molecules in silica dust exposure rats; however, the determination of the molecules regulating Th1 or Th2 cells requires further study.

## 4 Discussion

Exogenous antigens enter the human respiratory system and induce inflammatory and anti-inflammatory responses that can result in immune dysfunction including tolerance of autoantibodies and ambient particles as well alteration of the ability to respond to exogenous pathogens and microorganisms.^35,36^ Multifunctional DC cells play a central role in innate and adaptive immunity upon pathogen exposure, and they capture, process and present antigens, activate other immunocytes and eliminate debris and other materials.^37,38^ In the present study, we detected the number of AMs and DCs in rat lung tissue over 84 days by flow cytometry. The results demonstrated that the number of AMs significantly decreased, whereas DCs showed the opposite trend during the inflammation period of silicosis. These results were in accordance with the results reported by Beamer, who demonstrated that the percent and absolute number of AMs decrease significantly with concomitant significant increase in DCs.^39^ This suggested that compared with AMs, DCs are more tolerant when attacked by silica dust and may have primary role in identification, uptake and presentation of silica dust to activate T lymphocytes. Subsequently, during fibrosis, AMs and DCs both increased significantly, participating in fibrosis to create and sustain a profibrotic lung microenvironment^40,41^. In addition, our observation time was much longer than that of Beamer, and a longer period was beneficial for determining the complete trends of AMs and DCs.

Polarization of Th1/Th2 cells and cytokines has been confirmed in immunity-related diseases,^42,43^ and many studies have confirmed that it also plays an important role in the process of fibrosis.^44–46^ To date, most silicosis models have only detected Th1/Th2 cells and cytokines within 28 days.^47^ However, the occurrence and development of silicosis is a gradual and prolonged process, and the entire process of silicosis cannot be reflected in 28 days. Therefore, we dynamically examined Th1/Th2 cells and IFN-γ and IL-4 cytokines for up to 84 days. The results showed that compared with the observations for the control, the polarization of Th1 cells and IFN-γ expressions significantly increased during the inflammatory stage in silica exposure dust rats and that the polarization of Th2 cells and IL-4 expressions significantly increased during the fibrotic stage of silicosis. It was demonstrated that the Th1 cells were predominant and activated in the inflammation period of silicosis and secreted large amounts of IFN-γ to inhibit fibrosis; in fibrosis during silicosis, the Th2 cells were predominant and activated, and they secreted IL-4 to promote pulmonary fibrosis. Our findings are consistent with Wu's study of INF-γ dominance (Th1 cytokine) in pigeon breeder's lung patients at the acute/sub-acute stage and IL-4 dominance (Th2 cytokine) at the chronic stage after pulmonary fibrosis occurred.^48^

Recent studies have found that DCs can regulate polarization of Th1/Th2 cells in a variety of diseases, but this regulation in silicosis is rarely reported.^49–51^ Then, we detected CD80, CD86, MHC-II and IL-12 expressions to confirm whether these molecules produced by DCs can initiate Th cell polarization in silica dust exposure rats. The results suggested that DCs presented silica dust to T cells *via* elevated expressions of CD80, CD86, MHC-II and IL-12 and thus regulated the polarization of Th1/Th2 cells in silica dust exposure rats. However, the determination of the specific cytokine that regulates Th1 or Th2 cells during such exposure still needs to be researched.

In summary, our study demonstrated that DCs accumulated in lung tissues of silica dust exposure rats and regulated the polarization of Th1/Th2 cells *via* CD80, CD86, MHC-II and IL-12 expressions, indicating that DCs may play a critical role in modulating immune homeostasis during silicosis in rats. However, the detailed mechanism of DCs regulating the polarization of Th1/Th2 cells remains to be further investigated.

## Author contributions

CF and WY designed the study. LB, SN, JW, and LZ conducted experiments, analyzed the data, and drafted the manuscript. All authors read and approved the final manuscript.

## Conflicts of interest

None of the authors has a financial relationship with a commercial entity that has an interest in the subject of this manuscript.

## Supplementary Material
